# Proceedings of the Addiction Health Services Research (AHSR) 2020 Virtual Conference: Part 1

**DOI:** 10.1186/s13722-020-00207-5

**Published:** 2020-12-30

**Authors:** Sara J. Becker

**Affiliations:** Center for Alcohol and Addictions Studies, Brown University School of Public Health, 121 South Main Street, Providence, Rhode Island 02912 USA

The Addiction Health Services Research (AHSR) Conference is an annual event that brings together researchers, clinicians, policymakers, community partners, and funders to advance the latest, cutting-edge addiction health services research. Health services research has been broadly defined as a multidisciplinary field of scientific inquiry that studies how organizational structures and processes, financing systems, health technologies, social factors, and personal beliefs and behaviors affect access to addiction services, as well as the utilization, quality, and cost of health care [1]. Ultimately, such research has the potential to improve the addiction health services system in a way that improves the health and well-being of persons with or at risk of substance use disorders.

Annual AHSR conferences were first offered over a decade ago in response to a perceived need for a formal venue to disseminate addiction health services research, promote networking of addiction health services scholars, and advocate for addiction health services research funding [2]. The conferences are not supported by a formal organization or society, but rather are supported by a National Planning Committee comprised of representatives from various universities, contract research organizations, funding entities, and individual investigators. Members of the committee alternate hosting the annual conference. AHSR 2020 was chaired by Sara Becker, Ph.D., of the Brown University School of Public Health with Sean Grant, Ph.D., of Indiana University Richard M. Fairbanks School of Public Health serving as co-chair and poster session moderator. Activities were formally coordinated by the New England Addiction Technology Transfer Center, a training and technical assistance center funded by the Substance Abuse and Mental Health Services Administration (SAMHSA), and the Center for Alcohol and Addiction Studies at the Brown University School of Public Health. Additional funding support came from the National Institute on Drug Abuse, grant R13DA044722, awarded to Randall Brown, Ph.D., and Andrew Quanbeck, Ph.D.

The 2020 AHSR Conference was scheduled to be held in Providence, Rhode Island, from October 14th to October 16th. In response to the COVID-19 pandemic, the 2020 conference was held fully virtually. Guided by the 2020 AHSR Planning Committee, the strategic decision was made to offer an abbreviated conference experience at no cost to attendees as a public service. The abbreviated conference retained several core elements of AHSR including a pre-conference workshop for early career researchers, four plenary sessions featuring nationally renowned addiction health services researchers, AHSR Investigator Awards for early career researchers, opportunities for mentorship, and virtual poster presentations.

A key goal of AHSR 2020 was to offer an open source experience. Posters accepted to AHSR 2020 were disseminated in multiple ways. First, all posters were shared through the Open Science Framework: each poster had a unique page that presented the poster authors, title, abstract, and a PDF of the full poster available for downloads (visit the conference page at https://osf.io/meetings/AHSR2020/). Presenters were also encouraged to upload an audio recording of the presentation. Second, hyperlinks to the Open Science Framework pages were available in the AHSR electronic brochure (available for download via ahsrconference.org). Third, all of the accepted posters were compiled into virtual slideshows, which were shared in between conference sessions. Fourth, the posters were disseminated via social media via a new AHSR twitter page (@AHSRConference). Finally, all of the abstracts will be published in this supplemental journal issue.

The AHSR 2020 Conference began on October 14, 2020 with a pre-conference workshop titled “Publishing in Addiction Health Services Research,” led by Kelly Dunn, Ph.D., M.B.A., and Lewei Allison Lin, M.D. This pre-conference workshop was followed by a top-rated poster session. The top-rated poster session was facilitated by Sean Grant, Ph.D., and featured live presentations of nine of the posters that received the highest overall scores from blind peer reviewers. A total of 144 individuals engaged in activities on this day.

On October 15, 2020, AHSR activities included four AHSR Investigator Award presentations, two plenary sessions, and another top-rated poster session. The day began with presentations from the first four AHSR Investigators—Morgan Shields, Ph.D., Benjamin Shepherd, M.Ed., Jenny Zhen-Duan, Ph.D., and Jesse Hinde, Ph.D.—each of whom shared their award-winning work. Next, in the first plenary session, Laura Kwako, Ph.D., and Tisha Wiley, Ph.D., described funding priorities from the National Institute on Alcohol Abuse and Alcoholism (NIAAA) and the National Institute on Drug Abuse (NIDA), respectively. Time was also spent answering questions from the audience about specific funding initiatives and opportunities. The second plenary session, titled “Accelerating the Research to Practice Pipeline via Hybrid Trials,” was then moderated by Geoffrey Curran, Ph.D. Attendees heard from three panelists about their experiences conducting hybrid trials: Hildi Hagedorn, Ph.D. (hybrid type 1), Bryan Garner, Ph.D. (hybrid type 2), and Mark McGovern, Ph.D. (hybrid type 3). Ana Baumann, Ph.D., a health equity and implementation science expert, served as Discussant and challenged the panelists to think about how to center the voices of underserved populations in their work. The day closed with a second top-rated poster session featuring another set of 10 posters with the highest overall scores. Two hundred sixty-three individuals engaged in conference activities on October 15th.

The final day of the conference, October 16, 2020, consisted of two AHSR Investigator Award presentations and two plenary sessions. Activities were initiated with presentations from the final two AHSR Investigators—Manuel Cano, Ph.D., and Hillary Samples, Ph.D. These presentations were followed by a plenary session titled, “Point/Counterpoint: It’s Time to X the Waiver vs. Patients with Opioid Use Disorders Deserve Trained Providers,” which was facilitated by H. Westley Clark, M.D., M.P.H., J.D. Arguments were heard in favor of X-ing the buprenorphine waiver by Sarah Wakeman, M.D., and against X-ing the waiver by David Fiellin, M.D. Each presenter shared their initial argument and had a chance to share a rebuttal, before engaging in a lively question and answer with conference attendees. In the final session, Ayana Jordan, M.D/Ph.D., provided an inspiring talk titled “Developing an Anti-Racist Recovery Movement in Research and Practice.” Her session was again followed by an interactive question and answer session. This closing session was attended by 207 unique individuals.

Throughout the conference sessions, the Brandeis-Harvard mentoring program provided opportunities for attendees to be mentored or to mentor other individuals involved in the Addiction Health Services Research field. AHSR 2020 had about 60 attendees sign up to participate in the mentoring program. Mentors and mentees were encouraged to schedule virtual sessions either during the AHSR conference or in the weeks following. As in prior years, Maureen Stewart, Ph.D. and Hillary Richards, MSc, MA of the Brandeis-Harvard NIDA Center to Improve System Performance of Substance Use Disorder Treatment (P30 DA035772; Principal Investigator: Constance Horgan, Sc.D.) led the matching of mentees with mentors and the administration of the program.

In total, 368 unique individuals engaged in AHSR 2020 programming. About 60% of conference attendees identified as early career researchers. Of those who attended, 25 applied for an AHSR Investigator Award and six were ultimately selected based on the strength of their poster, written statement, and CV. There were 172 posters submitted to AHSR 2020, of which 137 were accepted. Posters are presented in this supplement according to their track. Specific track titles include: AHSR Investigator Award Winners (AW); Top-Rated Posters (TR); Health Disparities and Health Equity (HD); Systems-Wide Interventions (SW); Treatment Development and Evaluation (TD); Models and Methods (MM); and Implementation Science (IS).

AHSR 2020 was truly a collaborative, team effort. Multiple colleagues at the Brown School of Public Health and the New England ATTC were instrumental to the success of the conference. Core team members included (in alphabetical order) Sarah Helseth, Ph.D., Patience Moyo, Ph.D., Sharon Lang, B.A., Raymond Sanchez, B.A., Kelli Scott, Ph.D., Mika Salas, B.S., and Julia Yermash, B.S.

The AHSR 2020 Planning Committee also played a vital role in the development and success of the conference, by providing strategic direction, reviewing poster abstracts, and reviewing award applications. In addition to the core team members listed above, committee members included Amanda Abraham, University of Georgia; Andrea Acevedo, Tufts University; Jenny Becan, Texas Christian University; Geoffrey Curran, University of Arkansas for Medical Sciences; Bri Deyo, University of Wisconsin-Madison; Lori Ducharme, National Institute on Drug Abuse; Pete Friedmann, Baystate Health; Mitchell Garets, University of Utah; Bryan Garner, RTI International; Joseph Glass, Kaiser Permanente; Adam Gordon, University of Utah; Heather Gotham, Stanford University; Bryan Hartzler, University of Washington; Constance Horgan, Brandeis University; Aaron Johnson, Augusta University; Hannah Knudsen, University of Kentucky; Laura Kwako, National Institute on Alcohol Abuse and Alcoholism; Kathryn McCollister, University of Miami; Sean Murphy, Cornell University; Hillary Richards, Brandeis University; Sharon Reif, Brandeis University; Angela Robertson, Mississippi State University; Maureen Stewart, Brandeis University; Tisha Wiley, National Institutes of Health; and Emily Williams, University of Washington.

Feedback from AHSR 2020 was overwhelmingly positive, with respondents indicating that the virtual format was “inclusive,” “engaging,” “well-organized,” “professional,” and “seamless,” with plenary talks that were “important,” “high quality,” and just the “right length.” Materials from AHSR 2020, including video recordings of all of the conference sessions, remain freely available via the conference website (ahsrconference.org). Individuals interested in participating in future AHSR conferences should email ahsr2020@brown.edu to be added to the conference mailing list.
